# Supplementary material for: Fermentation Products Originated from Bacillus subtilis Promote Hepatic–Intestinal Health in Largemouth Bass, Micropterus salmoides
Source: Biology (Basel). 2025 Jun 2;14(6):646. doi: 10.3390/biology14060646 (PMC12189297; doi:10.3390/biology14060646)
Supplement: Supplementary file 1 [file biology-14-00646-s001.zip › biology-3569654-supplementary.pdf]

biology

MDPI

Supplementary Table S1

Effect of different temperature, pH and cation on the FP (Inhibition zone diameter, mm)

Condition		*Aeromonas veronii*	*Aeromonas hydrophila*	*Aeromonas salmonicida*
Temperature	Control (30)	12.74	11.03	11.42
	20	12.51	11.59	11.33
	40	12.46	11.32	11.37
	60	12.41	11.01	12.18
	80	12.17	11.55	11.94
	100	0	0	0
PH	Control (7)	12.55	13.76	12.12
	3	13.14	14.74	14.73
	4	12.86	15.1	14.51
	5	11.46	13.8	13.19
	6	12.3	12.95	13.82
	8	13.59	13.29	12.11
	9	12.21	11.7	11.94
	10	12.68	12.14	12.23
	11	12.33	12.63	12.2
NaCl	Control (0)	13.77	14.04	14.77
	50mmol/L	14.31	13.85	14.66
	100mmol/L	14.03	14.17	14.85
	150mmol/L	14.56	14.68	14.12
	200mmol/L	14.87	14.76	14.28
$MgCl_2$	Control (0)	12.8	12.62	15.25
	50mmol/L	13.33	13.6	14.29
	100mmol/L	14.78	13.96	14.75
	150mmol/L	14.14	13.46	15.47
	200mmol/L	13.69	13.02	14.42
$CaCl_2$	Control (0)	13.73	11.51	15.33
	50mmol/L	14.88	13.28	14.75
	100mmol/L	14.69	13.63	14.67
	150mmol/L	15.62	13.21	16.58
	200mmol/L	15.77	13.05	16.66
KCl	Control (0)	12.83	14.43	13.28
	50mmol/L	13.22	14.5	13.31
	100mmol/L	13.48	14.09	14.23
	150mmol/L	14.08	15.04	14.17
	200mmol/L	13.97	15.11	14.18
